# Cardiac interstitial tetraploid cells can escape replicative senescence in rodents but not large mammals

**DOI:** 10.1038/s42003-019-0453-z

**Published:** 2019-06-13

**Authors:** Kathleen M. Broughton, Tiffany Khieu, Nicky Nguyen, Michael Rosa, Sadia Mohsin, Pearl Quijada, Bingyan J. Wang, Oscar H. Echeagaray, Dieter A. Kubli, Taeyong Kim, Fareheh Firouzi, Megan M. Monsanto, Natalie A. Gude, Robert M. Adamson, Walter P. Dembitsky, Michael E. Davis, Mark A. Sussman

**Affiliations:** 1 San Diego State University Heart Institute and the Integrated Regenerative Research Institute, 5500 Campanile Drive, San Diego, CA 92182 USA; 2 Cardiovascular Research Center, Temple University, 3500 N. Broad St., Philadelphia, 19140 PA USA; 3 Division of Cardiology, Sharp Memorial Hospital, 8010 Frost St., San Diego, 92123 CA USA; 4 Biomedical Engineering and Medicine, Emory University, 1760 Haygood Dr., Atlanta, 30322 GA USA

**Keywords:** Heart stem cells, Heart stem cells

## Abstract

Cardiomyocyte ploidy has been described but remains obscure in cardiac interstitial cells. Ploidy of c-kit+ cardiac interstitial cells was assessed using confocal, karyotypic, and flow cytometric technique. Notable differences were found between rodent (rat, mouse) c-kit+ cardiac interstitial cells possessing mononuclear tetraploid (4n) content, compared to large mammals (human, swine) with mononuclear diploid (2n) content. *In-situ* analysis, confirmed with fresh isolates, revealed diploid content in human c-kit+ cardiac interstitial cells and a mixture of diploid and tetraploid content in mouse. Downregulation of the p53 signaling pathway provides evidence why rodent, but not human, c-kit+ cardiac interstitial cells escape replicative senescence. Single cell transcriptional profiling reveals distinctions between diploid versus tetraploid populations in mouse c-kit+ cardiac interstitial cells, alluding to functional divergences. Collectively, these data reveal notable species-specific biological differences in c-kit+ cardiac interstitial cells, which could account for challenges in extrapolation of myocardial from preclinical studies to clinical trials.

## Abstract (web summary)

Broughton et al. report differences between rodent and large mammals with respect to ploidy of the cardiac interstitial cell population. These species-specific differences in ploidy of cardiac interstitial cells suggest potential challenges in extrapolating myocardial preclinical studies from rodent to large mammals.

## Introduction

Ploidy, the number of chromosomes within a cell, is maintained as diploid sets in most mammalian somatic tissues. Although diploid chromosome content is typical, higher levels of ploidy with multiple chromosome sets referred to as polyploid are widespread across phyla as well as diverse organisms, including mammals. Polyploidy first observed in plants over a hundred years ago is an advanced evolutionary trait of adaptation and survival^1,2^, evidenced by polyploid genomic content in over 70% of flowering plants^3^. Polyploidization is often a consequence of normal development, aging, disease, and tissue regeneration processes^4,5^. Polyploid DNA content in mammals normally arises during fetal development in the placenta (trophoblasts)^6^ or in postnatal development of the liver (hepatocytes)^5,7^, bone marrow (megakaryocytes)^8^, skin (karatinocytes)^9^, and during aging and disease within the liver and heart (cardiomyocytes)^5,7,10^. Generation of polyploid chromosome content occurs through initiation of abortive mitotic activity, leading to increased chromosome content within a single nucleus (endoreplication) or generation of multiple nuclei (endomitosis)^11,12^. Biological significance of ploidy variations investigated for decades has spawned substantial speculation, yet the fundamental biological impetus for genome multiplication remains surprisingly obscure.

Decades of research uncovered many instances where polyploidization is intimately linked to biological processes. Polyploidization creates genetic diversity enabling adaptation to environmental stress^13^. Ploidy influences cellular biological properties in multiple ways, and the presence of polyploid cells in mammalian organisms indicates evolutionary conservation consistent with a fundamental biological role^5,12,14^. Several beneficial traits have emerged to account for initiation of polyploidization including adaptation to environmental stress, cell cycle regulation, DNA damage resistance, abrogation of senescence and apoptosis, tissue repair, and regeneration^5,7^. Many of these examples occur in plants and invertebrate species where regenerative capabilities are biologically normal (plants regenerate from cuttings, planaria can be cut into half and regenerate^1,2^). Similarly, many lower vertebrates with remarkable regenerative properties possess polyploid DNA content (salamanders, fish, and frogs^1,4^) or genome duplication (zebrafish^15^). Perhaps not coincidentally, regenerative tissues in the adult human body, including liver, skeletal muscle, and skin, all possess polyploid cellular populations.

Although polyploidy is characteristic of mammalian myocardium, the adult heart is notoriously refractory to de novo cardiomyogenesis that correlates with increasing ploidy content^16,17^. Indeed, cellular and nuclear ploidy in adult mammalian cardiomyocytes is in a dynamic state, dependent upon genetics, age, and environmental circumstances^10,16,18,19^. Binucleated cardiomyocytes are generally unresponsive to proliferation stimuli, perhaps serving a role in maintaining cell function and/or adaptation to stress^13^. The fluid nature of ploidy in adult mammalian heart suggests functional involvement, albeit poorly understood, in response to injury and aging. Surprisingly, despite abundant recognition of ploidy as an inherent biological property of the adult mammalian cardiomyocytes, ploidy status of the cardiac nonmyocyte population and biological significance of ploidy in myocardial interstitial cells has received relatively little attention.

The cardiac interstitial cell (CIC) population is a heterogeneous collection of cell types, including (but not limited to) fibroblasts, stromal cells, and various progenitor/stem cell populations^20,21^. The CIC population serves a critical role in both homeostasis as well as response to injury, although ploidy shifts in CICs remain essentially unstudied. Among CIC cell types, c-kit+ CICs proliferate in response to infarction injury by transient proliferation^22^. The impact of myocardial infarction injury upon CICs, including that of c-kit+ cells, has been extensively studied in exquisite detail for decades, as summarized in many research studies and reviews^23–26^. Based upon our extensive experience studying c-kit+ cells^21,22,27–30^, we focused this investigation upon understanding of ploidy in the c-kit+ subset of CICs. Functional consequences of polyploidy in these interstitial cells may correlate with transcriptional changes, leading to genetic diversity, adaptation to injury, and improved survival in stress response. Mouse mesenchymal stem cells (mMSCs) can exist as tetraploids and cultured tetraploid mMSCs demonstrate reduced p53 activity compared with diploid mMSCs^31^. In our laboratory, mMSCs fused with mouse cultured ckit+ CICs, termed cardiac stem cells (CSCs), resulted in chimeric stem cells (CardioChimeras) with sustained enhanced support for functional repair after infarction injury compared with either parent line^32^. In reviewing these studies, we noticed that CardioChimeras with higher chromosome counts correlated with better functional capacity in vitro and in vivo. The CardioChimera study prompted further investigation of CSC ploidy differences between rodents, large mammals, and humans due to striking differences between species.

Polyploid cells in the CIC population implicate a previously unappreciated aspect of heterogeneity that could account for observations of remarkable myocardial reparative potential in murine experimental model systems. Profound CIC expansion and acute remodeling to reinforce ventricular wall integrity after massive infarction injury is a survivable and tolerable event in rodents, whereas similar levels of damage end in death or rapid adverse remodeling in larger mammals. As polyploid cells correlate with specialized functions in tissues, this study centers upon DNA and ploidy content within a select subpopulation of cardiac nonmyocyte cells. Multiple rodent (mouse, rat), large mammal (feline, swine), and human samples were studied over development, adulthood, and after pathologic injury, using in situ, in vitro and freshly isolated cell preparations. The results provide compelling evidence for a previously uncharacterized cardiac-specific tetraploid nonmyocyte interstitial cell population in rodents, as well as a role for ploidy to influence physiologic response to environmental stress and pathological injury.

## Results

### Frequency of higher ploidy in human cardiomyocytes increases with age and disease

**Fig. 1:**
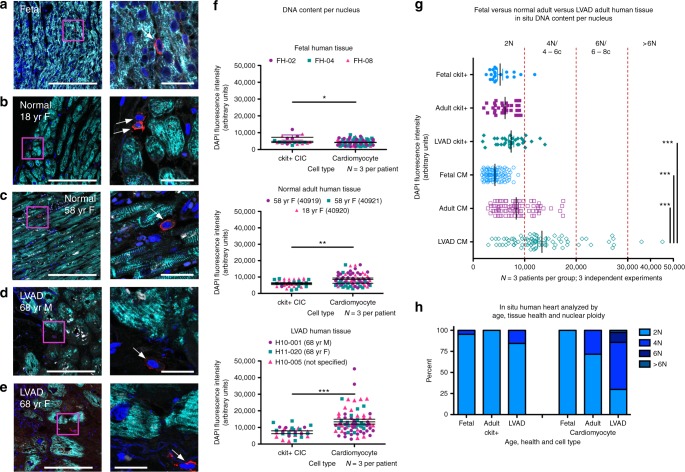
Higher ploidy level increases with age and disease in human cardiomyocytes. Human myocardial tissue sections assessed by in situ quantitation of ploidy level from 16-week-old fetal (**a**), 18- (**b**), and 58- (**c**) year-old female with normal heart histology and function, and 68-year-old male (**d**) and female (**e**) explanted from the left ventricle free wall from heart failure patients receiving the LVAD implant (zoomed-out scalebar = 150 μm; zoomed-in scalebar = 25 μm). Boxed regions of higher magnification are shown to the right of each scan. Arrows point to example cCICs included in the analysis. Quantitation of in situ DNA content per nucleus of cardiomyocytes and ckit+ cells measured by DAPI fluorescent intensity of the nucleus within 3D reconstruction of tissue, analyzed by *t* test (**f**). Compiled data of in situ DNA content per nucleus of cardiomyocytes and ckit+ cells measured by DAPI fluorescent intensity of the nucleus within 3D reconstruction of tissue (**g**). Percent of cCICs and cardiomyocyte nuclei with diploid tetraploid and higher ploidy content (**h**). *P < 0.05; **P < 0.01; ***P < 0.001. Data are presented as mean±SEM and analyzed using a *t* test (**f**) or one-way ANOVA with Bonferroni post hoc test (**g**)

Ploidy is increased in adult human cardiomyocytes isolated from heart failure patients relative to normal control samples^33^. In situ ploidy analysis was performed through quantification of diamidino-2-phenylindole (DAPI) fluorescence intensity of reconstructed confocal z-stacks^34^. Ploidy of single nuclei from cCICs and cardiomyocytes was assessed by in situ DAPI fluorescence intensity of reconstructed confocal z-stacks from fetal (Fig. 1a), normal adult (Fig. 1b, c), and heart failure patients (Fig. 1d, e). Ploidy grouping was determined by frequency of cells within fluorescence intensity ranges and results reported in aggregate from multiple patients and experiments (Fig. 1f). Diploid content was predominant in cCICs from fetal, normal adult, and LVAD adult tissues. Cardiomyocytes in fetal samples demonstrate a diploid content, whereas normal adult cardiomyocytes are ~71% diploid with 29% tetraploid and LVAD adult cardiomyocytes demonstrated the greatest variation in ploidy content with ~30% diploid, 56% tetraploid, and 14% greater than tetraploid (Fig. 1g, h). These results demonstrate less ploidy variation with aging or disease in ckit+ CICs compared with cardiomyocytes in the human heart.

### Multiple ploidy populations in murine cardiomyocytes and c-kit+ CICs emerge during postnatal stages to adulthood

**Fig. 2:**
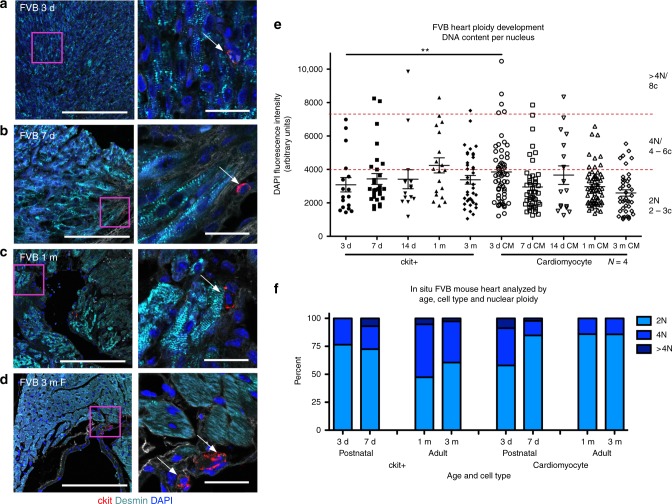
Ploidy variability in murine cardiomyocytes and c-kit+ CICs. FVB mouse myocardial tissue sections assessed by in situ quantitation of ploidy level post birth at 3 days (**a**), 7 days (**b**), 30 days (**c**), and 90 days (**d**) (zoomed-out scalebar = 150 μm; zoomed-in scalebar = 25 μm). Boxed regions of higher magnification are shown to the right of each scan. Arrows point to example cCICs included in the analysis. Compiled quantification of in situ DNA content per nucleus of cardiomyocytes and ckit+ cells measured by DAPI fluorescent intensity of the nucleus within 3D reconstruction of tissue (**e**). Percent of cCICs and cardiomyocytes nuclei with diploid tetraploid and higher ploidy content (**f**). **P < 0.01. Data are presented as mean ± SEM and analyzed using a *t* test, at each time point, or one-way ANOVA with Bonferroni post hoc test within cell types (**e**)

Ploidy content within mononucleated adult murine cardiomyocytes is predominantly mononuclear diploid, transitioning to binucleation during development^16^. Postnatal proliferation of murine cardiomyocytes and CICs occurs primarily within the first week after birth^35^, as cardiomyocytes acquire variation in ploidy, but ploidy of the CICs remained obscure. Ploidy of cCICs and cardiomyocytes was assessed by in situ DAPI fluorescent intensity analysis at postnatal ages of 3 days, 7 days, 1 month, and 3 months (Fig. 2). cCICs demonstrated ~75% diploid content at 3 and 7 days post birth, with 47 and 61% diploid at 30 and 90 days post birth, respectively. Single nuclei of cardiomyocytes display ~58% diploid content at 3 days post birth, which became ~85% diploid by day 7 postnatal and maintained at that level at 1 and 3 months (Fig. 2e, f). Cardiomyocytes undergo DNA doubling and a proliferative burst during the first few days after birth^36^, consistent with an increased number of cardiomyocytes possessing tetraploid nuclei at 3 days post birth. These results demonstrate that adult cCIC are diploid, tetraploid with low-frequency higher ploidy cells and ploidy transition of single nuclei in cardiomyocytes from neonate to adult postnatal development.

### Tetraploid content of endogenous ckit+ CICs uniquely observed the murine heart

**Fig. 3:**
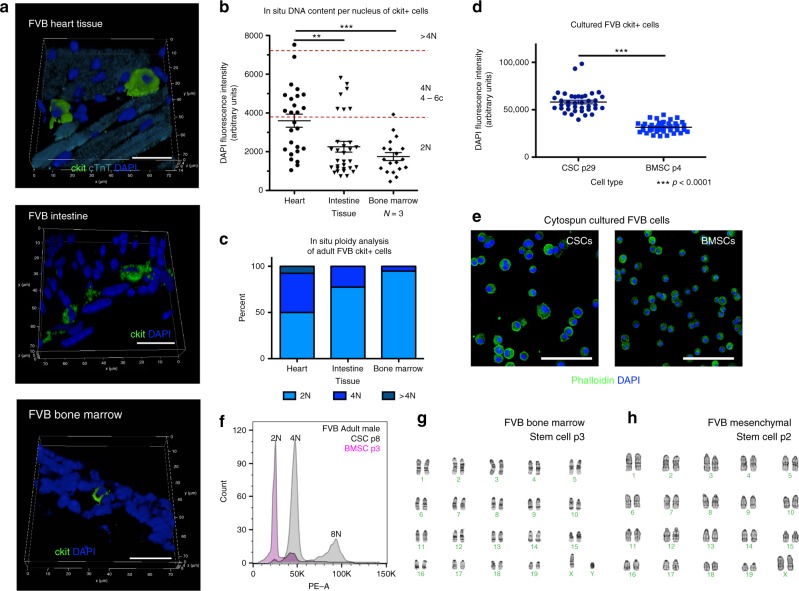
Tetraploidy is characteristic of an endogenous c-kit+ CIC subpopulation. FVB mouse myocardial, intestine, and bone marrow tissue sections assessed by in situ quantitation of ploidy level at 90 days post birth (scalebar = 20 μm) (**a**). Compiled quantification of in situ DNA content measured by DAPI fluorescent intensity of the nucleus within 3D reconstruction of tissue demonstrates that cardiac ckit+ CICs have higher ploidy levels compared with ckit+ from intestine or bone marrow (**b**). Percent of ckit+ cells nuclei with diploid tetraploid and higher ploidy content from myocardial, intestine, and bone marrow tissue (**c**). Ckit+ cells isolated and cultured from cardiac tissue demonstrate higher ploidy levels compared with cultured ckit+ cells from bone marrow (**d**), measured by DAPI fluorescent intensity of the nucleus within 3D reconstruction of tissue (scalebar = 150 μm) (**e**) and propidium iodine fluorescent intensity of the nucleus using flow cytometry (**f**). G-band karyotype analysis verifies diploid content of cultured ckit+ bone marrow stem cells (**g**) and bone marrow-derived mesenchymal stem cells (**h**). **P < 0.01; ***P < 0.001. Data are presented as mean±SEM and analyzed using a *t* test (**d**) or one-way ANOVA with Bonferroni post hoc test (**b**)

**Fig. 4:**
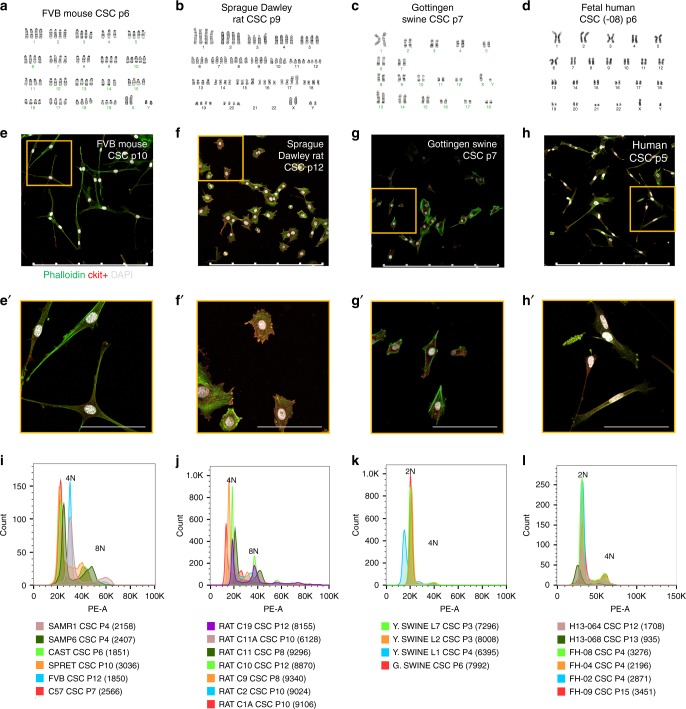
Mononuclear tetraploid content distinguishes rodent cardiac stem cells in vitro. G-band karyotype analysis performed upon cultured CSCs reveals tetraploid content of rodent mouse (**a**) and rat (**b**) in contrast to diploid content of swine (**c**) and human (**d**) samples. Immunocytochemistry of CSC verifies mononuclear content of karyotyped cells (scalebar = 500 μm) (**e**–**h**) and zoomed-in images (scalebar = 100 μm) (**e**′–**h**′). Multiple samples from different CSC lines verify consistent tetraploid content in adult mouse (**i**) and rat (**j**) CSCs, while swine (**k**) and human (**l**) CSCs are consistently diploid. Cell counts included in flow-cytometry analysis provided in parenthesis

Ploidy state of ckit+ cells within adult FVB mouse heart, intestine, and bone marrow tissues was determined by in situ DNA analysis. Ploidy of mononuclear cCICs shows both diploid and tetraploid cells, whereas diploid content predominates within ckit+ cells of the intestine and bone marrow (Fig. 3a–c). C-kit+ CICs and BMSCs were isolated, expanded, and assessed for ploidy after culturing, as our prior study reveals substantial transcriptional reprogramming following culturing of stem cells^21^ (cultured ckit+ CICs referred to as CSCs). Marked divergence in ploidy levels was evident in CSC cultures relative to BMSCs of adult FVB mice showing tetraploid versus diploid content, respectively, by confocal (Fig. 3d, e) and flow- cytometry (Fig. 3f) analyses. Karyotyping verified CSC mononuclear tetraploid (Fig. 4a) versus diploid content of BMSCs and bone marrow-derived MSCs (Fig. 3g, h). Collectively, these findings highlight distinct tetraploid content of c-kit+ CIC and CSCs relative to c-kit+ cells from non-cardiac murine tissue sources or cultured BMSCs.

### Rodent CSCs acquire uniformly mononuclear tetraploid content in vitro, in contrast to diploid cells of pig or human origin

Additional evidence to support aforementioned ploidy findings was derived from characterization of cell cultures expanded from human, large animal, and rodent samples. Six distinct laboratory mouse strains (FVB, C57/B6, CAST, SPRET, SAMP6, and SAMR1) were used for CSC isolation and expansion, as well as multiple clonal expanded lines from Sprague Dawley rat samples. Human CSCs were isolated from fetal, pediatric, and LVAD patient cardiac tissue. Large animal CSCs were represented by Gottingen and Yorkshire swine, as well as domestic feline samples. Karyotyping reveals mononuclear tetraploid content in rodent CSCs (Fig. 4a, b and Supplementary Fig. 1A, B), whereas human, swine, and feline CSCs are mononuclear diploid (Fig. 4c, d and Supplementary Fig. 1C–F). Mononuclear content per cell was verified by immunocytochemistry (Fig. 4e–h, Supplementary Fig. 2A–O). Furthermore, karyotype findings were consistent with flow-cytometry analyses (Fig. 4i–l). These results reinforce distinct mononuclear tetraploidy in rodent CSCs versus typical diploid content found in human and large mammalian cells, both in situ and in vitro.

### CSC ploidy correlates with proliferative capacity

Morphologic and proliferative characteristics of CSCs were assessed in vitro. Cell surface area and major to minor axis ratio are similar within each species (Supplementary Fig. 2P–S). Likewise, proliferation rates for each species assayed were comparable (Supplementary Fig. 2T–W). Replicative potential between human and murine CSCs was assessed with continuous passaging until senescence-mediated arrest. Proliferation ceases between passages 12 and 26 for fetal human CSCs and between passages 9 and 15 for adult human CSCs isolated from heart failure patients. In contrast, replicative senescence was never achieved with mouse or rat CSCs, with proliferative capacity extending beyond 50 passages. In comparison, mouse BMSCs cease proliferation between passages 4 and 6. Average growth rate and doubling time for human and standard lab mouse strain (FVB, C57) CSCs demonstrate similar profiles for the first 3 days after plating, with differences only found at day 4 (Supplementary Fig. 3A, B). Morphology and ploidy of FVB CSCs remained stable between early and late passages (Supplementary Fig. 4A–D), while proliferation rate increased with early long-term passaging and then leveling off between passages 50 and 100 (Supplementary Fig. 4E, F). Cell cycle-related genes analyzed using RT-PCR reveal upregulation of *Ccna2, Ccnb2*, and *Cdc25c* in murine CSCs comparing passages 11 versus 100 (Supplementary Fig. 4G). Increased Cdc25c expression in high-passage CSC was confirmed by immunoblot (Supplementary Fig. 4H). Collectively, these results establish differences in proliferative capacity with tetraploid CSCs escaping typical replicative senescence arrest normally observed in diploid rodent BMSCs and human CSCs.

### Human and mouse CSCs express senescence-associated p53-associated markers

Tetraploid cells ordinarily senesce in response to endoreplication error^37^, but tetraploid mCSCs have escaped this checkpoint. Cellular senescence markers include p53 (*Trp53*), p16 (*Cdkn2a*), and p21 (*Cdkn1a*)^38^. P53 is associated with the inhibition of cellular growth and subsequent apoptosis; mdm2 controls p53 cell-cycle arrest through mdm2–p53 interaction^39^. To understand the continued growth of tetraploid mCSCs but senescence of hCSCs, a gene panel array of senescence markers and protein was assessed in hCSCs and mCSCs at low- versus high-passage points (Supplementary Fig. 5A, B). Expression of p53 pathway genes was analyzed by RT-PCR and proteins were measured by immunoblot. Genes upregulated in the high-passage compared with low-passage hCSCs include the apoptosis marker *BCL2* and endothelial growth factor receptor *KDR* (Supplementary Fig. 5A), while genes upregulated in the high-passage compared with low-passage mCSCs include *Cdk2* to regulate S-G2 cell cycle and anti-apoptosis marker *Hspb1* while *Cdkn1a, Cdkn2a,* and *Mdm2* are downregulated (Supplementary Fig. 5B). In terms of protein, high-passage hCSCs express increased total p53 and p53 phosphorylated at Serine 15 (Supplementary Fig. 5C), known to activate p53^39,40^, while MDM2 remains constant at low and high passages (Supplementary Fig. 5D). In mCSCs, protein expression of total p53, phosphorylated p53 at Serine 15, and MDM2 is decreased in high versus low passage (Supplementary Fig. 5E, F). Collectively, these data provide insights into the cell-cycle and p53-associated senescence regulation of murine CSCs that evidently override p53-mediated cell-cycle arrest.

### Murine CSCs increase negative p53 feedback loop with increased passaging

Tripartite motif-containing (TRIM) superfamily proteins regulate pathogen-recognition and transcriptional pathway defense^41^ by p53 potentiation (TRIM 13,19) or inhibition (TRIM 25,28,29) via the Mdm2–p300–p53 complex^42,43^. TRIM proteins are known to regulate multiple cellular processes, including proliferation, differentiation, development, autophagy, and apoptosis in mammalian cells^44,45^. Analysis of TRIM expression in hCSCs at replicative senescence revealed increased TRIM 13 transcription, together with decreased TRIM 25 and 28 transcription and protein expression (Supplementary Fig. 6A, C, D). Conversely, high-passage mCSCs show transcriptional inhibition for TRIM 13 and 19, with concomitant increases in TRIM 25, 28, and 29 (Supplementary Fig. 6B). Protein expression for each TRIM family member was downregulated in high-passage mCSCs (Supplementary Fig. 6E, F) and may result as high-passage mCSCs that demonstrate decreased total p53 and p53 phosphorylated at Serine 15 (Supplementary Fig. 5B). TRIM 29 transcription increased in both human and murine CSCs at higher passage, and further studies would be necessary to determine the significance of this finding. These data provide additional evidence that p53 is present in both hCSCs as well as mCSCs at higher-passage points, but could be subject to TRIM-mediated transcriptional influences upon activity level.

### Freshly isolated murine cardiac lin– c-kit+ CIC exhibit ploidy variation

**Fig. 5:**
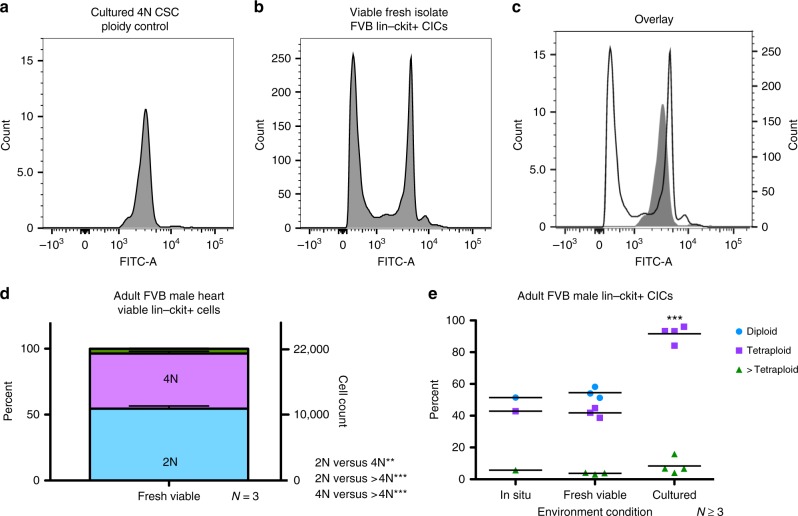
Polyploid states are present in freshly isolated murine c-kit+ CICs. Hematopoietic lineage negative, c-kit positive CICs were freshly isolated and sorted for viability and ploidy content from FVB adult male mice. Using the cultured FVB CSC as a tetraploid control (**a**), fresh isolates of Lin– ckit+ CICs exhibit a mixture of diploid and tetraploid cells (**b**) shown by an overlay of tetraploid control versus fresh isolate sample (**c**). Lin–ckit+ CICs possess a similar percentage and cell count distribution of diploid and tetraploid cells with a small fraction of cells with ploidy greater than tetraploid, statistically analyzed using one-way ANOVA (**d**). Summary of polyploid state from FVB mouse lin–ckit+ cells of the heart, with compiled in situ and freshly isolated cells, revealing a mixture of mononuclear diploid and tetraploid levels, while cultured CSCs are tetraploid; all groups demonstrated a small fraction of tetraploid cells. **P < 0.01; ***P < 0.001. Data are presented as mean ± SEM and analyzed using one-way (**d**) or two-way (**e**) ANOVA with Bonferroni post hoc test

Ploidy analysis of freshly isolated hematopoietic lineage (lin)– ckit+ viable CICs was performed using Vybrant dyecycle green sorted by flow cytometry. Tetraploid CSCs, verified with karyotype (Fig. 4a), were used as a 4N control (Fig. 5a) for determination of gating for freshly isolated, viable, FVB lin–ckit+ ploidy populations. Freshly isolated cCIC exhibit two primary ploidy populations (Fig. 5b) compared with tetraploid-cultured mCSC (Fig. 5c, overlay). Viable, freshly isolated cCIC are a mixture of diploid (54.51 ± 3.47%), tetraploid (41.8 ± 3.04%), and higher ploidy levels (3.69 ± 0.56%) (Fig. 5d; 22,000 ± 2915 cells analyzed per experiment). Collectively, mixed ploidy states are comparable for in situ and fresh isolation determinations, whereas cultured murine c-kit+ CICs are uniformly tetraploid (Fig. 5e). These results demonstrate mixed ploidy states in endogenous murine c-kit+ CICs and culture conversion of c-kit CICs into a pure tetraploid population.

### Population subsets of diploid versus tetraploid murine fresh isolate lin– c-kit+ CICs revealed by single-cell transcriptional profiling

**Fig. 6:**
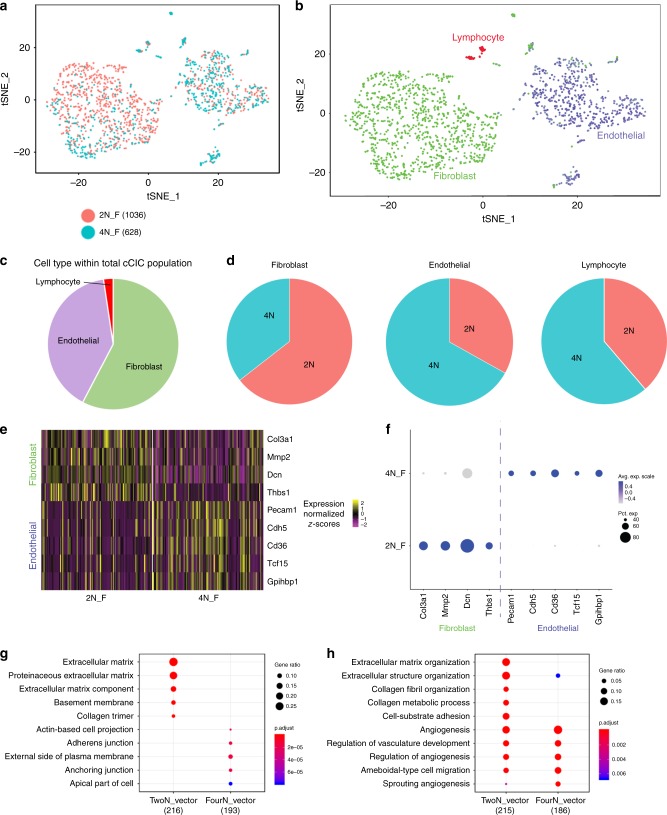
Distinct population characteristics of diploid versus tetraploid fresh murine Lin–c-kit+ isolates revealed by single-cell RNA sequencing (scRNA-Seq). Freshly isolated, viable diploid and tetraploid Lin–ckit+ CICs from adult FVB mouse were analyzed using scRNA-Seq. The diploid population (salmon) predominately cluster together, while tetraploid cCICs (teal) cluster in a different cell group (**a**; cells analyzed per group identified next to ploidy state). Identification of the cell populations demonstrates that cCICs are a heterogeneous population of fibroblast, endothelial, and lymphocyte cells (**b**). Percent of cCICs based on cell type (**c**). Percent of each cell type based on ploidy content (**d**). Heatmap of upregulated differentially expressed genes (DEGs) specific to endothelial and fibroblast markers confirm transcriptional differences in murine-derived diploid (2N_F) and tetraploid populations (4N_F) (**e**). These DEGs are also displayed in frequency and expression level between the diploid and tetraploid cCICs (**f**). The top ten gene ontology (GO) terms upregulated in the 2N population display extracellular matrix cellular components, while the 4N population cellular component is junction oriented (**g**). The top ten GO terms by biological process upregulated in the 2N population represent both extracellular matrix and angiogenesis processes, while the 4N population is primarily angiogenesis oriented (**h**)

Single-cell RNA sequencing (scRNA-Seq) reveals transcriptome differences within freshly isolated CICs^20^, as well as between fresh isolated cCICs and cultured CSCs^21^. Although subtle, distinctions between diploid versus tetraploid fresh isolates of cCICs were revealed through scRNA-Seq transcriptional profiling. Multidimensional reduction processing coupled with t-Distributed Stochastic Neighbor Embedding (t-SNE) representation through Seurat R package and Barnes–Hut tree-based algorithm reveals three primary aggregates within the diploid and tetraploid viable cells (Fig. 6a), consistent with interstitial/fibroblast, identified with *Col3a1*, endothelial, identified with *Cd36*, and lymphocyte, identified with *Cd3e* (Fig. 6b, c). Interestingly, these three subgroups were differentially distributed in diploid and tetraploid fresh isolates for fibroblast (69% vs. 38%), endothelial (29% vs. 59%), and lymphocyte (2% vs. 3%) (Fig. 6d).

Comparing gene expression profiles between diploid and tetraploid populations, 223 differentially expressed genes (DEGs) were identified in the diploid population versus 187 DEGs in the tetraploid population, as shown for the top fibroblast and endothelial DEGs (Fig. 6e, f and Supplementary Table 1). Extracellular matrix (ECM) and fibroblast gene expression frequency was prominent in the normalized diploid population (Supplementary Fig. 7A, B), identified with *Col3a1* (65.72% 2N vs. 34.28% 4N); *Mmp2* (65.26% 2N vs. 34.74% 4N); *Dcn* (55.22% 2N vs. 44.78% 4N); and *Thbs1* (69.27% 2N vs. 30.73% 4N). Fibroblast gene expression per cell, shown with violin plots, was elevated in the diploid population (Supplementary Fig. 7C). Endothelial gene expression frequency was higher in the normalized tetraploid population (Supplementary Fig. 8A, B), identified with *Pecam1* (34.23% 2N vs. 65.77% 4N); *Cdh5* (33.73% 2N vs. 66.27% 4N); *Cd36* (35.26% 2N vs. 64.74% 4N); *Tcf15* (44.82% 2N vs. 55.18% 4N); and *Gpihbp1* (37.5% 2N vs. 62.5% 4N). Violin plots of each gene demonstrate higher endothelial expression per cell within the tetraploid population (Supplementary Fig. 8C). Analysis of the gene onthology (GO) by cellular component verified that the 2N population is uniquely linked to ECM-related components (Fig. 6g) and biological processes (Fig. 6h) compared with the 4N population. These data collectively indicate that the majority of 2N lin–ckit+ freshly isolated CICs from murine hearts are fibroblasts related to the ECM, while the majority of fresh isolate 4N lin–ckit+ CICs from murine hearts are endothelial related to support angiogenesis.

### Ploidy reduction occurs in murine lin– c-kit+ CIC following infarction

**Fig. 7:**
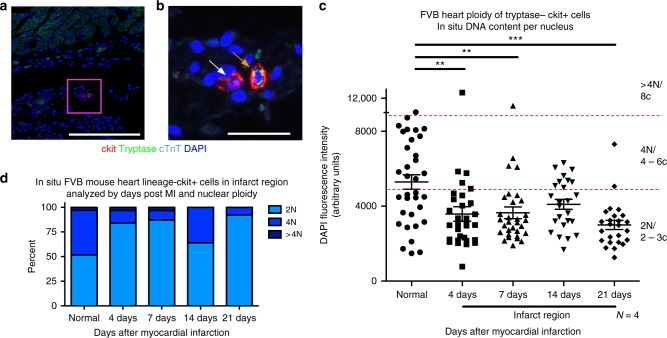
Reduction of DNA content in Lin– c-kit+ CICs following myocardial infarction. Quantitation of in situ nuclear DNA content in adult FVB mice following myocardial infarction measured by DAPI fluorescent intensity of tryptase–, ckit+ CIC nuclei. 3D reconstruction of cardiac tissue was performed at 4, 7, 14, and 21 days post injury in the infarction zone. Normal, age, gender, and strain-matched hearts were used as a control. Tryptase+, a marker of mast cells, was only found in injured hearts within the first week after injury and was not included in the analysis, as shown with tryptase–, ckit+, and tryptase+ ckit+ CICs in the infarction zone of an adult, 7-day post-MI FVB heart (**a**, scalebar = 150 μm) with a boxed region of higher magnification (**b**, scalebar = 25 μm). The white arrow identifies cCICs included in the analysis and the orange arrow identifies a mast cell, not included in the study. Compiled dot plot of in situ DNA content per nucleus of lin–ckit+ cells demonstrates higher frequency of diploid levels in the infarction region at all time points post MI (**c**), and is verified upon quantification of percent diploid, tetraploid, and greater than tetraploid content (**d**). **P < 0.01; ***P < 0.001. Data are presented as mean ± SEM and analyzed using one-way ANOVA with Bonferroni post hoc test (**c**)

The cCIC population expands in response to infarction injury^22,46^. Ploidy changes within the cCIC population in response to myocardial infarction were assessed in situ in the infarction zone using immunohistochemistry, with non-infarcted hearts as control samples. Mast cells identified in infarcted hearts using the surface marker tryptase were excluded from analysis (Fig. 7a, b). Ploidy of cCIC within the infarction and border zone region was over 80% diploid at 4 and 7 days after myocardial infarction, with 64% diploid at day 14 post MI and 92% by day 21 post MI (Fig. 7c) compared with control. These results demonstrate that ploidy of cCICs in the infarction zone is predominantly diploid, trending toward fibroblast phenotype.

## Discussion

Polyploidy is an advanced evolutionary trait that facilitates regeneration^1^ and capability to cope with stress through possession of extra DNA copies or alternate gene sets. Polyploid cells are present throughout human lifespan, leading to the assertion that acquisition of polyploid DNA content is an adaptive process to avoid cell death as well as oncogenic transformation^47,48^. Regenerative activity and functional specialization of cells in mammals are associated with polyploid cell populations^49,50^. In addition, ploidy levels can be influenced as a consequence of aging, stress, acute injury, and environmental conditions^1,2,11,13^. Thus, ploidy exists in a dynamic and regulatable state consistent with and essential for normal biological function^12,13^. Ploidy variation in cardiomyocytes has been studied for decades, through binucleation or mononuclear endoreplication, associated with postnatal development, oxidative damage, and telomere erosion^51^. Increased metabolic activity with increased protein production could also drive cardiomyocyte polyploidy, as demonstrated in smooth-muscle cells and megakaryocytes^52,53^. To our knowledge, previous ploidy studies of myocardial biology focused upon cardiomyocytes^5,10,16–19,54^, whereas assessment of CIC ploidy has not been addressed, leading to this report that provides several important insights regarding cCIC ploidy content.

A straightforward conclusion from our findings is species and tissue-specific correlation of tetraploid cCICs in the murine myocardium. Starting with ploidy analysis of in situ cardiomyocytes and cCICs performed in rodent (mouse, rat), large mammal (feline, swine), and human samples, association of tetraploid DNA content with murine myocardial samples was consistently demonstrable from in situ, in vitro, and fresh isolation experiments (Figs. 2–5). Ploidy state of human cardiomyocyte is a complex, variable, and fluid condition requiring detailed analyses with more patients and sample points, similar to conclusions from published literature. Previous myocardial ploidy studies in humans limited to cardiomyocytes show over 40% mononuclear polyploids by adulthood^55^ and up to 80% polyploids in patients with infarction injury^56^, similar to our findings (Fig. 1). In murine cardiomyocytes, rodent binucleation increases dramatically between postnatal day 4 and 12^57^ and by adulthood, over 80% of single cardiomyocyte nuclei are diploid^58^. Recently, our group published that the peak of cardiomyocytes in S/G2/M cell-cycle phase occurs at day 2 of postnatal development^59^. Our study indicates that ~40% of postnatal day 3 single-nucleus DNA content of cardiomyocytes are polyploid, reducing to 15% by day 7 and maintained at 15% into adulthood (Fig. 2). This study complements findings for myocyte ploidy from prior studies, although limitations of our in situ study include analysis based on a limited number of cells and presumption of ploidy content remaining comparable throughout the heart, since determinations were made using adult human left ventricle free-wall samples versus the entire heart for mouse samples. Analysis of regional ploidy will determine if differences exist between ventricular walls and septum. Although our analysis focused upon determinations for DNA quantitation within individual nuclei rather than nucleation state, future studies need additional discrimination of multinucleated versus mononuclear cells to determine cellular ploidy status. This issue of cardiomyocyte cell-cycle state and ploidy is the focus of ongoing studies and we look forward to exploring these ideas in future investigations.

Tetraploidy in rodent CSCs was confirmed by characterization of cultured rat CSCs from the Davis laboratory (Fig. 4). Human cCICs are a diploid population from development through adulthood and in pathological states. In comparison, rodent cCICs exhibit mononuclear diploid and tetraploid population heterogeneity. Therefore, profound differences in DNA content exist between cCICs of human and large mammal versus rodent origin, with the reasonable possibility that this fundamental biological difference between species could account, at least in part, for challenges in extrapolating findings from murine preclinical studies to larger mammals, including humans.

Infarction injury in the murine heart favors expansion of diploid cCICs in the infarct/border zone. Diploid cCIC predominance may arise from proliferation of diploid cells, ploidy reduction from tetraploid to diploid, or a combination of these events. In Drosophila intestine, polyploid intestinal stem cells (ISCs) undergo amitosis to regenerate new diploid ISCs in response to environmental stress^60^. Likewise, hepatocytes undergo ploidy reduction during liver regeneration after hepatectomy^61^. In myocardium, fibroblasts are activated, proliferate, and infiltrate in response to myocardial infarction^62^. Considering that the majority of diploid cCICs display a fibroblast transcriptome profile, cCICs within the infarction are likely fibroblasts. Ongoing studies to delineate CIC ploidy changes in response to infarction are underway.

Functional in vitro differences were consistent, regardless of species between diploid and tetraploid CSCs. Human CSCs from heart failure patients undergo replicative senescence at passages 12–16, whereas pediatric and fetal CSCs typically expand through 21–26 passages. The respective ploidy states of human and rodent CSCs remained invariant throughout passaging, showing stability of divergent ploidy states between the two species. Prior in vitro studies involving human and mouse embryonic stem cells (ESCs) demonstrate polyploidy resulting from failed mitosis^63^. We find that mouse ckit+ BMSCs maintain diploid content after culturing, while mCSC acquire pure tetraploid content upon culturing, although initially isolated as a mixture of diploids and tetraploids. An important distinction to emerge from in vitro rodent ckit+ CIC studies was culture adaptation, leading to uniform tetraploidy of CSCs capable of escaping replicative senescence, whereas diploid CSCs disappear from the amplified population. Multiple concurrent processes could explain tetraploid dominance in vitro. Examples include the diploid population undergoing endoreplication or endomitosis to become tetraploid, indirect influence of tetraploids to inhibit diploid proliferation through cell–cell communication, possibly via secreted factors, or greater adaptive capacity of the tetraploid population to the stress of in vitro expansion, leading to diminished competitive capability of the diploids. Indeed, tetraploid cCICs and CSCs may inhibit diploid proliferation through cell–cell communication, as recently discovered between tetraploid and diploid hepatocytes to maintain stability and prevent tumor formation^64,65^. Although we cannot exclude fusion as a possible mechanism^66^, no evidence of fusion was observed in time-lapse recordings of CSC cultures (unpublished observation). Understanding the in vitro ploidy shift of mCSC population may provide mechanistic insights into replicative senescence shown in hCSCs and mBMSCs.

On a molecular level, rodent CSCs show a profile consistent with inactivation of cell-cycle checkpoints, together with downregulation of p53 that could be functionally inhibited by differential expression of TRIM family members. The relationship of p53 to suppression of tetraploidy in murine embryonic development showed p53 downregulation rescuing tetraploid embryos^67^ and providing chromosomal stability for tetraploid cells^68^. Also, p53 plays a role in ploidy resolution during liver regeneration^69^. Furthermore, assessment of DNA content in mesenchymal stem cells derived from p53 knockout mice cultured for 40 days showed conversion to tetraploidy in contrast to normal control cells that remained diploid^70^. It is tempting to speculate that tetraploid CSCs represent an evolutionary adaptation of CICs providing a functional benefit to rodents. The capacity of CICs to avoid cellular senescence without oncogenic transformation would provide a reservoir of cells with enhanced proliferative potential as well as resistance to environmental stress and preservation of functional responsiveness. Downregulation of p53 in high-passage mCSCs is coupled with transcriptional upregulation of negative feedback regulation of p53 with TRIM 25, 28, and 29. Proteins for these TRIM isoforms are down in high-passage mCSCs, which could result as p53 protein also down in high-passage mCSCs. Of course, mRNA transcript levels do not necessarily correlate with protein expression^71^, as was the case here with select TRIM isoforms for the human and mouse CSCs. Additional studies revealing how various TRIM isoforms respond to environmental stress are clearly warranted to delineate functional, molecular, and mechanistic differences between diploid versus tetraploid CSC that may reveal how rodent hearts withstand pathological damage, whereas larger animals may be not as well equipped.

CIC number increases through postnatal proliferation as well as post infarction in rodents^35,56,72^ and humans^56^. These early studies assessed CIC population as a single-cell type, but recent advent of scRNA-Seq technology reveals heterogeneity of the CIC population normally present in the adult murine heart^17,20^. Findings at the transcriptome level point to not only cellular heterogeneity, but also a blend of subtle transcriptional profiles within each cell subtype that reflects population diversity^20,21^. In comparing the fresh isolate 2n and 4n hematopoietic lineage-negative, ckit-positive CICs, the results demonstrate differences in the heterogeneity of the two populations. The 2n and 4n freshly isolated lineage– ckit+ CICs from adult murine hearts exhibit differential DEG expression, identifying the 2N cells predominately as fibroblast and ECM-support cells, while the 4N population is endothelial and angiogenesis-support cells (Fig. 6). Interestingly, lineage–ckit+ CICs in the infarction zone of an adult mouse heart are nearly all diploid, supporting remodeling of the heart and scar formation with some vascularization (Fig. 7).

Ploidy changes are postulated as a rapid and potentially reversible response to environmental stress^73–75^. Multiple explanations have been offered for the biological significance of polyploidy, including the intriguing idea that polyploidy appears to be permissive for recovery from the senescence-arrested state^76^ and overcomes replication stress-induced senescence^77^. Specific to mammals, some cell types are consistently polyploid across species, while others possess variable amounts of DNA dependent upon the species and the environmental stress^13^. Hepatocytes are polyploid in rodents, large animals, and humans^5,7^ and undergo DNA reduction in response to environmental stress to regenerate the liver^78^. Although the mechanisms of DNA reduction are not well understood, aneuploidy to increase genetic diversity and resistance to chronic liver injury occurs frequently in human (30–90%) and mice (60%)^61,79^. Whereas aneuploidy can sometimes be maladaptive, leading to oncogenesis or cell death, polyploidy generally allows for preservation of normal cellular function^80,81^. Tetraploidy can arise when a proliferating cell fails to undergo cytokinesis following DNA replication, but in general this primarily leads to cell-cycle arrest^82^. In rare cases, a select polyploid cell can escape treatments intended to promote senescence, such as chemotherapeutic treatment intended to arrest tumor progression^83^. The relevance for polyploidy as a mechanism for avoiding senescence and preserving cell survival in vivo is the subject of ongoing controversy in the cancer literature^76,77^. Likewise, additional studies are necessary to understand the mechanism differences of ploidy states in the CIC population.

In the cardiovascular system, polyploidy is a normal condition of both vascular smooth-muscle cells^84^ and cardiomyocytes, where an increased frequency of binucleation in rodents is observed compared with increased DNA content within a single nucleus in large mammals and humans^54^. Variation in cardiomyocyte ploidy between species has been documented^34,54^. Higher frequency of diploid cardiomyocytes correlated with better physiologic recovery from myocardial infarction in a survey of multiple murine strains^16^. Diabetic stress also influenced the ploidy level of cardiomyocytes^85^. Although increased numbers of CICs in response to infarction are evident in mammalian models, only one prior publication examined ploidy of the non-myocyte population and concluded that human CICs are diploid^86^, consistent with our finding that human cCICs are diploid in situ and upon isolation and expansion. While polyploidy is a relatively common occurrence in human cardiac muscle and vasculature, human CICs do not readily adopt polyploidy as is the case for rodents.

The importance of the CIC population in response to aging and pathologic injury is well accepted, but only recently have studies delved further into a subtle difference distinguishing subpopulations. This report is the first to our knowledge delineating ploidy of CICs rather than cardiomyocytes in the heart. Focusing upon the cCIC subpopulation allowed for selection of a small subset of the total CIC pool that presumably represents a cell type with progenitor-like characteristics relative to the entire CIC population, including multiple committed cell types. While the ultimate role of polyploidy in the CIC population remains to be determined in future studies, findings in this report establish the benchmark of distinct DNA content differences between rodent and large mammals. We hypothesize that an endogenous response within rodent mammals lacking in larger mammals contributes to the blunting of injury along with enhanced capacity for repair^87^. Future studies regarding fresh isolate human lineage– ckit+ CICs may provide additional insights into differences between endogenous human and rodent samples. The challenge of defining functional differences between the 2n and 4n ploidy states is complicated by the inherent plasticity of ploidy state: attempts to isolate and separately expand diploid versus tetraploid mouse CSCs were unsuccessful, because diploid cells underwent binucleation and growth arrest, allowing tetraploid cells to persist. Although at present, we have not performed a direct diploid-to-tetraploid comparison of CSCs from the same species to determine in vitro and in vivo adoptive transfer functional activity, our findings provide a compelling rationale for investigations to incorporate polyploidization into cardiac regenerative medicine by induction of human CSC tetraploidization as a method to enhance functional activity.

## Methods

### Mouse CSC and BMSC isolation

All procedures and experiments involving mice were conducted by observing ethical guidelines for animal studies, as approved by the SDSU Institutional Animal Care and Use Committee. Mouse CSCs were isolated from both male and female mice for initial karyotype and ploidy observations and maintained as previously described^88^. Cultured male FVB CSCs were used at passages 11 and 100 for experiments unless otherwise stated. Bone marrow stem cells (BMSCs) were isolated from 12-week-old male and female FVB mice by flushing the femur and tibiae with 5% fetal bovine serum in PBS through a 40-µm filter and centrifuged (10 min, 600 × *g*, 4 °C) for initial ploidy analysis with experiments performed on male FVB BMSC for a consistent control against mCSCs. Cells were suspended in 96-well U-bottom plates with media consisting of STEMSPAN, 1% PSG, Flt-3, and SCF: 50 ng/ml and IL-3, IL-6, and TPO: 10 ng/ml. The media was changed every 2 days and cells were used at passages 2–4 for experiments unless otherwise stated.

### Human CSC isolation

Samples were received from patients with informed consent at Sharp Hospital, San Diego, CA under the Sharp Hospital institutional review board (IRB) approval following NIH guidelines for human subjects’ research. Human CSCs were isolated and maintained as previously described^89^. Karyotype was performed on both male and female CSCs. Cultured hCSCs from three different human tissue samples were used at passage 5 and the respective higher senescence passage (p. 14–24, dependent on sample) for experiments unless otherwise stated.

### Cell karyotype

All cells were G-banded and karyotyped by *Cell Line Genetics* (Madison, WI), except for Sprague Dawley Rat CSCs (karyotyped by Emory University Hospital Oncology Cytogenetics Laboratory, Atlanta, GA) and Feline CSCs (karyotyped by KaryoLogic, North Carolina Biotech Center, Durham, NC). Cells were maintained according to a laboratory protocol, until prepared for live-cell 1-day delivery shipping according to an identical preparation protocol by a service provider (e.g., see https://www.clgenetics.com/wp/wp-content/uploads/2014/06/Mailing-Live-Cultures-4.17.14.pdf).

### Proliferation assay and cell-doubling time

Cell proliferation was determined using the CyQuant Direct Cell Proliferation Assay (Life Technologies, Carlsbad, CA) according to the manufacturer’s instruction and as previously described^90^. Population-doubling times were calculated using the readings from CyQuant Direct Proliferation Assay and use of a population-doubling time online calculator (http://www.doubling-time.com/compute.php).

### Flow cytometry

Flow cytometry to measure DNA content in terms of fluorescence intensity is well documented^91,92^. Suspended cells were fixed using 70% ethanol and kept at −20 °C for at least 24 h prior to measuring DNA content using propidium iodine (PI) with RNAse (BD Biosciences, San Jose, CA). Approximately 50,000 cells were centrifuged at 1300 rpm for 5 min at 22 °C; the ethanol was removed, and the cells were resuspended in 200 μL of PI and kept at 37 °C in the dark for 10 min. In total, 10,000 events per sample were recorded with doublets and unstained events removed from analysis. PI fluorescence intensity was measured using a BD FACSCanto. The gating strategy is shown in Supplementary Fig. 9. To sort fresh isolate cells by DNA content, cells are labeled with Vybrant DyeCycle Stain (ThermoFisher, Carlsbad, CA) at a ratio of 1:60,000 in 300 μL of wash buffer (PBS with 0.5% BSA) at 37 °C in the dark for 10 min. Cells are sorted based on FI of Vybrant dye, using a BD FACSAria II, sorting at 9 μL/s. Data are analyzed using FLOWJO (Ashland, OR).

### Immunocytochemistry

Stem cells were placed at a density of 10,000 per well of a two-chamber permanox slide and were fixed using 4% paraformaldehyde for 10 min at room temperature. Cells were permeabilized using 0.03 M glycine for 5 min, followed by 0.5% Triton X-100/PBS for 10 min to reduce non-specific binding. Cells were then blocked with TNB for 30 min at room temperature, followed by a primary antibody in TNB labeling overnight at 4 °C. The next day, cells were rinsed with PBS and then labeled with secondary antibody and/or phalloidin in TNB for 1.5 h. Subsequent tyramide amplification was performed as necessary. The cells are then rinsed with PBS, DNA is stained with 1 mg/μl 4′,6-diamidino-2-phenylindole (DAPI) (ThermoFisher, Carlsbad, CA) 1/10,000 in PBS for 5 min, and Vectashield mounting medium is applied (Vector Labs, Burlingame, CA). Slides were visualized using a Leica TCS SP8 confocal microscope. Primary and secondary antibodies used, as well as concentrations, are listed in Supplementary Table 2.

### Immunohistochemistry

Mouse hearts were retroperfused, removed from the animal, and fixed in 10% formalin overnight. Tissue samples from other organs or heart sections from larger mammals were also formalin fixed overnight. Tissue was then treated with 70% ethanol prior to paraffin embedding, using a Leica eASP300 enclosed tissue processor (Leica Biosystems, Buffalo Grove, IL). Paraffin-processed tissues were cut into 5-micron sections and slide mounted using a HM 355S Automatic Microtome (Thermo Fisher Scientific, Waltham, MA). Heart sections were deparaffinized and incubated with primary and secondary antibodies as previously described^93^. Subsequent tyramide amplification was performed as necessary. The cells are then rinsed with PBS, DNA is stained with 1 mg/μl 4′,6-diamidino-2-phenylindole (DAPI) (ThermoFisher, Carlsbad, CA) 1/10,000 in PBS for 5 min, and Vectashield mounting medium is applied (Vector Labs, Burlingame, CA). Slides were visualized using a Leica TCS SP8 confocal microscope. Primary and secondary antibodies used are listed in Supplementary Table 2. DAPI concentration was determined by consistency of results, based upon known diploid content per nuclei (Supplementary Fig. 10).

### In situ confocal microscopy imaging

Measuring fluorescence intensity of the DAPI signal is a known protocol to measure a relative quantity of DNA^94,95^. Briefly, the cells were imaged with a Leica SP8 confocal microscope and a 40× water Objective (Leica, Buffalo Grove, IL), maintained with a 1 Airy unit pinhole size. The DAPI wavelength excitation is 405 and the two-hybrid-photomultiplier tube (HyD) emission bandwidth was set at 412–452 nm, at a power intensity of 1.5 W and gain of 150 V. The CENPB probe is tagged with a Cy3 fluorophore, excited at 561 nm, with an emission bandwidth set at 571–620 nm under a power intensity of .25 W and gain of 60 V. The format of the acquisition was 1024 × 1024 at 400 Hz. The zoom for locating ckit+ cells in tissue was kept at 1, until located. All cells scanned, in both tissue and cell culture were zoomed at a factor of 4, to create a pixel dimension of 0.071 μm in both the x and y plane and 0.426 μm in the z-plane during the z-stack scanning. Five line averages and a single-frame average occurred in a bidirectional manner at a frame rate of 0.773/s. Each stack dimension was kept at an optimized system option, with a step size of 0.42 μm, with 19–25 slices included per z-stack. Z-stacks were then processed.

### Cytospun cell cultures

Cultured mCSCs and mBMSCs were suspended at 5 × 10^5^ cells/mL in PBS and 200 μL was loaded into EZ Cytofunnels (ThermoFisher, Carlsbad, CA) within a Shandon Cytospin 4 (ThermoFisher, Carlsbad, CA). Cells were spun down for 3 min on low acceleration and then fixed onto polylysine-coated slides using Shandon Cell-Fixx fixative (ThermoFisher, Carlsbad, CA). Cells were dried overnight and prepared for immunocytochemistry with phalloidin and DAPI at 1:10,000 for 5 min. Confocal imaging utilized a 63× oil objective (Leica, Buffalo Grove, IL), with zoom factor of 0.75, 1.1 Airy unit pinhole, and HyD emission bandwidth of 415–510 at power intensity of 1.5 W and gain of 150 V. The pixel dimensions were 0.241 μm in the x and y plane and 0.357 μm in the z-plane during z-stack acquisition; step size was optimum at 0.35 μm with 28–30 slices per z-stack.

### Z-stack analysis of DNA content

Z-stacks were reconstructed and analyzed using the Leica SP-8 software (Leica, Buffalo Grove, IL). To begin, a region of interest (ROI) was precisely drawn around the widest x–y dimension of each cell nucleus, with care taken to not over- or underdefine the ROI. Statistics of the z-stack ROI, the mean fluorescence intensity value of the processed pixels, and the ROI area, defined in pixel, are then multiplied to define a pure fluorescence intensity value for the ROI at the given dimension, and consistent gain and intensity. The fluorescence intensity (a unitless measurement) is reported as a relative quantity of DNA content per nucleus. The number of cCICs identified per in situ section varied, with analysis including 6–10 cCICs and 10–25 CMs per experiment. Human tissue was from a single section in the left ventricle, while murine tissue included all regions of the heart, and cells were selected based on identification of cCICs, which were located throughout the heart.

### Immunoblot sample preparation and experiment

CSC samples were collected in 1× sodium dodecyl sulfate (SDS) sample buffer with protease and phosphatase inhibitors. Cell lysates were boiled for 10 min and stored at −80 °C. The analysis for each protein was based on three or more separate immunoblots. Within each individual blot, samples were prepared from three or more independent cell lysates at low- and high- passage points. Each sample was normalized to the housekeeping protein GAPDH, and the three samples at low or high passage were averaged. Averaged protein quantities were statistically compared using a *t*-test analysis based on *N* = 3 separate experimental runs (except for human p53 and S15-phosphorylated p53, which was *N* = 4). Proteins were loaded into 4–12% Bis-Tris gel (Thermo Fisher Scientific, Waltham, MA) and run in 1× MES SDS running buffer (Thermo Fisher Scientific, Waltham, MA) at 150 V for 1.5 h on an electrophoresis apparatus (Invitrogen, Carlsbad, CA). Separate proteins were transferred to a polyvinylidene difluoride membrane in 1× transfer buffer (Thermo Fisher Scientific, Waltham, MA) and then blocked with Odyssey Blocking Buffer (TBS) (LI-COR, Lincoln, NE) for 1 h. After blocking, the membrane was incubated with primary antibodies in Odyssey Blocking Buffer at 4 °C overnight. The next day, the membrane was washed with 1× TBST three times at 15 min, room temperature on an orbital rocker. The membrane was then incubated with secondary antibodies in Odyssey Blocking Buffer for 1.5 h at room temperature on an orbital rocker, followed by three washes of 1× TBST for 15 min a wash, room temperature on an orbital rocker. The membrane was then scanned using an Odyssey CLx (LI-COR, Lincoln, NE). Primary and secondary antibodies used are listed in Supplementary Table 2.

### mRNA isolation, cDNA synthesis, and quantitative PCR

RNA was enriched using the Quick RNA Mini Prep kit from ZymoResearch according to the manufacturer instructions. Reverse transcriptase was performed using a protocol for the iScript cDNA Synthesis Kit (BIORAD, Hercules, CA). qRT-PCR was read after incubation of cDNA, primers (100 nM), and IQ SYBR Green Supermix (BIORAD). Data were analyzed using the ΔΔC(t). PCR Arrays for a generic cell cycle and p53 signaling analysis were purchased from BioRad (Hercules, CA). All other primer sequences were selected using primer-blast, verified through an oligo calculation, purchased from Thermo Fisher Scientific (Waltham, MA), and listed in Supplementary Table 3.

### scRNAseq cell preparation and sequencing

Preparation of cells for single-cell gene expression was performed according to manufacturer specification (10X Genomics, Pleasanton, CA). To optimize gem tagging efficiency, 2000 cells were loaded per group into the Chromium system and indexed sequencing libraries were constructed according to the manufacturer protocol, using the Chromium Sing Cell 3′ Library & Gel Bead Kit v2 (10X Genomics, Pleasanton, CA). Each library underwent quality control screening using a Bioanalyzer (Agilent Genomics, Santa Clara, CA) and quantified by quantitative PCR (KAPA Biosystems Library Quantification Kit for Illumina platforms) and Qubit 3.0 with dsDNA HS Assay Kit (Thermo Fisher Scientific, Watham, MA). Sequencing libraries were loaded at 2 pM on a Illumina HiSeq2500 with 2 × 75 paired-end kits, using the following read length: 98 bp Read 1, 8 bp i7 Index, and 26 bp Read 2.

### scRNAseq analysis

The raw data were processed with the Cell Ranger pipeline (10X Genomics; version 2.0). Sequencing reads were aligned to the mouse genome mm10. Preparations derived from two mouse hearts per sample were used to produce 1036 diploid and 628 tetraploid freshly isolated cells for analysis. Cells with fewer than 1000 genes or more than 10% of mitochondrial gene UMI count were filtered out and genes detected fewer than in three cells were filtered out^96^. Altogether, 1664 cells and 15,579 genes were kept for downstream analysis using Seurat R Package (v2.3.4). Approximately 2027 variable genes were selected based on their expression and dispersion. The first 12 principal components were used for the t-SNE projection^97^ and unsupervised clustering^96^. Gene expression pathway analysis was performed using clusterProfiler^98^.

### Myocardial infarction

Twelve-week-old female FVB were sedated and myocardial infarction was produced by ligating the left anterior descending (LAD) branch of the coronary artery using a 8-0 suture (Ethicon, Somerville, NJ). Echocardiography was performed under mild isoflurane sedation (0.5–1.5%) using a Vevo 2100 Linear Array Imaging ultrasound (FUJIFILM Visual Sonics, Toronto, ON, Canada). Cardiac function was analyzed in the parasternal long-axis view by tracking the endocardium with the supplied analysis software to obtain end-systolic volume, end-diastolic volume, ejection fraction, and heart rate. Noninvasive assessment of cardiac function in the parasternal long-axis view was performed at baseline, the day before surgery, and the day before harvesting to verify similar infarction size and cardiac function.

### Statistics and reproducibility

All data are expressed as mean +/− standard error of mean. Statistical analyses were performed within and between group comparisons, student *t* test, and one- and two-way ANOVA was applied with Bonferroni post hoc test, when applicable, using Graph Pad Prism v5.0 (GraphPad Software, La Jolla, CA). A value of <0.05 was considered statistically significant. Experiments were performed for both biological and technical replicates.

### Reporting summary

Further information on research design is available in the Nature Research Reporting Summary linked to this article.

## Supplementary information

Reporting Summary

Supplementary Information

## Data Availability

Sequence data that support the findings of this study have been deposited into the Gene Expression Omnibus with the primary accession code GSE122057, released on November 2, 2018.
