# Supplementary material for: Cardiac interstitial tetraploid cells can escape replicative senescence in rodents but not large mammals
Source: Commun Biol. 2019 Jun 13;2:205. doi: 10.1038/s42003-019-0453-z (PMC6565746; doi:10.1038/s42003-019-0453-z)
Supplement: Supplementary file 1 — Reporting Summary [file 42003_2019_453_MOESM1_ESM.pdf]

nature research

Corresponding author(s): Mark Sussman

Last updated by author(s): Mar 25, 2019

# Reporting Summary

Nature Research wishes to improve the reproducibility of the work that we publish. This form provides structure for consistency and transparency in reporting. For further information on Nature Research policies, see Authors & Referees and the Editorial Policy Checklist.

## Statistics

For all statistical analyses, confirm that the following items are present in the figure legend, table legend, main text, or Methods section.

| n/a | Confirmed | |
|---|---|---|
| ☐ | ☒ | The exact sample size (*n*) for each experimental group/condition, given as a discrete number and unit of measurement |
| ☐ | ☒ | A statement on whether measurements were taken from distinct samples or whether the same sample was measured repeatedly |
| ☐ | ☒ | The statistical test(s) used AND whether they are one- or two-sided<br>*Only common tests should be described solely by name; describe more complex techniques in the Methods section.* |
| ☒ | ☐ | A description of all covariates tested |
| ☐ | ☒ | A description of any assumptions or corrections, such as tests of normality and adjustment for multiple comparisons |
| ☐ | ☒ | A full description of the statistical parameters including central tendency (e.g. means) or other basic estimates (e.g. regression coefficient) AND variation (e.g. standard deviation) or associated estimates of uncertainty (e.g. confidence intervals) |
| ☐ | ☒ | For null hypothesis testing, the test statistic (e.g. *F*, *t*, *r*) with confidence intervals, effect sizes, degrees of freedom and *P* value noted<br>*Give P values as exact values whenever suitable.* |
| ☒ | ☐ | For Bayesian analysis, information on the choice of priors and Markov chain Monte Carlo settings |
| ☒ | ☐ | For hierarchical and complex designs, identification of the appropriate level for tests and full reporting of outcomes |
| ☒ | ☐ | Estimates of effect sizes (e.g. Cohen's *d*, Pearson's *r*), indicating how they were calculated |

*Our web collection on statistics for biologists contains articles on many of the points above.*

## Software and code

Policy information about availability of computer code

| | |
|---|---|
| Data collection | *Provide a description of all commercial, open source and custom code used to collect the data in this study, specifying the version used OR state that no software was used.* |
| Data analysis | *Provide a description of all commercial, open source and custom code used to analyse the data in this study, specifying the version used OR state that no software was used.* |

For manuscripts utilizing custom algorithms or software that are central to the research but not yet described in published literature, software must be made available to editors/reviewers. We strongly encourage code deposition in a community repository (e.g. GitHub). See the Nature Research guidelines for submitting code & software for further information.

## Data

Policy information about availability of data

All manuscripts must include a data availability statement. This statement should provide the following information, where applicable:

- Accession codes, unique identifiers, or web links for publicly available datasets
- A list of figures that have associated raw data
- A description of any restrictions on data availability

Gene expression pathway analysis was performed using clusterProfiler97. scRNA-Seq data generated was uploaded to the Gene Expression Omnibus (GEO submission GSE122057, released November 2, 2018).

# Field-specific reporting

Please select the one below that is the best fit for your research. If you are not sure, read the appropriate sections before making your selection.

☒ Life sciences ☐ Behavioural & social sciences ☐ Ecological, evolutionary & environmental sciences

For a reference copy of the document with all sections, see nature.com/documents/nr-reporting-summary-flat.pdf

# Life sciences study design

All studies must disclose on these points even when the disclosure is negative.

| | |
|---|---|
| Sample size | Sample size was dictated by performing a minimum of triplicate analyses with additional replicates as necessary to account for variability and achieve significance when warranted. |
| Data exclusions | No samples were excluded. |
| Replication | Each experimental protocol was replicated a minimum of three times with additional replicates as needed to achieve reproducibility. |
| Randomization | All samples were randomly selected and assigned to each group for analysis. |
| Blinding | Investigator was not blinded to the sample sets. |

# Reporting for specific materials, systems and methods

We require information from authors about some types of materials, experimental systems and methods used in many studies. Here, indicate whether each material, system or method listed is relevant to your study. If you are not sure if a list item applies to your research, read the appropriate section before selecting a response.

## Materials & experimental systems

| n/a | Involved in the study | |
|---|---|---|
| ☐ | ☒ | Antibodies |
| ☐ | ☒ | Eukaryotic cell lines |
| ☒ | ☐ | Palaeontology |
| ☐ | ☒ | Animals and other organisms |
| ☒ | ☐ | Human research participants |
| ☒ | ☐ | Clinical data |

## Methods

| n/a | Involved in the study | |
|---|---|---|
| ☒ | ☐ | ChIP-seq |
| ☐ | ☒ | Flow cytometry |
| ☒ | ☐ | MRI-based neuroimaging |

## Antibodies

| | |
|---|---|
| Antibodies used | *Describe all antibodies used in the study; as applicable, provide supplier name, catalog number, clone name, and lot number.* |
| Validation | Each commercially available antibody was validated by vendor. No proprietary, non-commercial antibody was used. |

## Eukaryotic cell lines

Policy information about cell lines

| | |
|---|---|
| Cell line source(s) | Cells were derived from primary tissue explands from hearts of mouse, rat, swine and human species. |
| Authentication | karyotype and flow cytometry were used to authenticate each line. |
| Mycoplasma contamination | Not tested for mycoplasma. |
| Commonly misidentified lines (See ICLAC register) | N/A |

## Animals and other organisms

Policy information about studies involving animals; ARRIVE guidelines recommended for reporting animal research

| | |
|---|---|
| Laboratory animals | Adult male and female mice of FVB, C57/B6, CAST, SPRET, SAMP6, and SAMR1 were randomly selected for initial ploidy analysis. FVB were selected for detailed tissue and MI studies. |

| | |
|---|---|
| Wild animals | N/A |
| Field-collected samples | N/A |
| Ethics oversight | Human samples were received from consenting patients with institutional review board (IRB) approval following NIH guidelines for human subjects' research. All procedures and experiments involving mice were conducted by observing ethical guidelines for animal studies as approved by the SDSU Institutional Animal Care and Use Committee. |

Note that full information on the approval of the study protocol must also be provided in the manuscript.

# Flow Cytometry

## Plots

Confirm that:

☒ The axis labels state the marker and fluorochrome used (e.g. CD4-FITC).

☒ The axis scales are clearly visible. Include numbers along axes only for bottom left plot of group (a 'group' is an analysis of identical markers).

☒ All plots are contour plots with outliers or pseudocolor plots.

☒ A numerical value for number of cells or percentage (with statistics) is provided.

## Methodology

| | |
|---|---|
| Sample preparation | For cell culture, cells were fixed in 70% ethanol overnight at -20C; cells were resuspended in propidium iodine for 15 minutes at 37C and then run on a FACSCanto. Live cells were stained with Vybrant CellCycle Dye 1:20,000 and then sorted on a FACSAria. |
| Instrument | BD FACSCanto and BD FACSAria |
| Software | Flow-Jo. |
| Cell population abundance | For cell culture, 10,000 events per sample were recorded |
| Gating strategy | Gating strategy was based on 2N mBMSCs and 4N mCSC, shown in figure 3. Cells positively stained for propidium iodine were analyzed for DNA content with doublets and unstained events removed from analysis. |

☒ Tick this box to confirm that a figure exemplifying the gating strategy is provided in the Supplementary Information.
